# A typology of subseasonal rainfall evolution during the southern Niger monsoon

**DOI:** 10.1371/journal.pone.0299771

**Published:** 2024-04-09

**Authors:** Colin P. Kelley, Shraddhanand Shukla, Kathryn Grace

**Affiliations:** 1 Department of Geography and Planning, Appalachian State University, Boone, NC, United States of America; 2 Climate Hazards Center, Department of Geography, University of California, Santa Barbara, Santa Barbara, CA, United States of America; 3 Minnesota Population Center, Department of Geography, Environment and Society, University of Minnesota, Twin Cities, Minneapolis, MN, United States of America; Universiti Teknologi Malaysia, MALAYSIA

## Abstract

Niger is highly vulnerable to rainfall variability, often with adverse socioeconomic consequences. This study examined observed subseasonal rainfall variability during Niger’s monsoon season (May to September). Using k-means clustering of dekadal (ten-day) rainfall, a typology was developed for the annual evolution of the monsoon season. Year-to-year rainfall variability for each of the first few dekads of the season is modest, but the middle, or peak of the rainy season demonstrates large interannual variability. Clustering analysis of annual timeseries for each dekad of the season revealed two types of monsoon progression. The distinction between the two types is strongly dependent on differences during the latter half of the season. For the first and third ten-day periods in August, and the first ten days in September, the two groups of years are more distinct. These results imply that while reliable prediction of the timing of anomalous onsets will be challenging, due to the relatively narrow range of uncertainty historically, there are opportunities for further exploration of dynamic and or statistical predictors or precursors using this typology that could potentially provide better information for decision-makers, especially with respect to agriculture.

## Introduction

Niger is highly vulnerable to a wide range of humanitarian challenges, including climate-related disasters, escalating conflicts and other socioeconomic concerns, all of which combine to adversely affect lives and livelihoods [1]. The country has a large and rapidly growing population (highest fertility rate in the world) that is strongly dependent (40% of total GDP) on rain-fed agriculture (millet, sorghum, cowpeas, groundnuts and other crops) and livestock as primary sources of subsistence and income [2–4]. Violence in and around the country has limited access to essential social services and aggravated chronic food insecurity and malnutrition. The region is still recovering from food crises brought about by severe droughts experienced in 2005, 2008, 2010 and 2012 and recent heavy rains and flooding in 2020 and 2021 [5]. In addition to the COVID-19 pandemic, Niger has faced frequent disease outbreaks such as cholera, measles, and meningitis, which are compounded by malnutrition and the effects of extreme weather events, especially floods and drought [1].

With constant challenges linked to environmental degradation, pervasive poverty and political instability, extremes of climate variability atop ongoing climate change will continue to exacerbate existing vulnerabilities in Niger. Water scarcity, increasing length of dry seasons and the impacts of higher temperatures could increase forced migration [6]. Water shortages caused by below‐average seasonal rainfall have negatively impacted the indigenous population, but irregular rainfall on subseasonal timescales, such as late or early onset of the monsoon, reduced duration of the rainy season, persistent dry or wet periods, or extremes of daily rainfall also deleteriously affect water and food security [4]. Late rains and prolonged drought episodes in Niger in 2004, 2009 and 2011 seriously compromised agricultural production and caused serious rangeland degradation, resulting in substantial losses of cereal, livestock and other assets in the pastoral and agro-pastoral zones, a drop in incomes, increased levels of household indebtedness, and deterioration of the nutritional situation [7]. Vulnerable populations have reacted by adopting strategies that threatened to affect social harmony and medium and long-term development, such as early transhumance with the risk of livestock invading crop areas before they have been harvested, destocking of animals, selling them off at poor prices, and excessive fuel wood extraction as an alternative source of revenue but leading to an acceleration of desertification [8].

In monsoon climates such as Niger, the magnitude of individual rainfall events is highly variable. A small number of extreme wet events in a given season, ranging from daily to dekadal timescales, can account for most of the total rainfall during the entire season. Seasonal forecasts of total precipitation and subseasonal forecasts (of week two and week three, for example) of total rainfall are currently being utilized [9–13].

Here we explore and propose a novel supplemental approach to forecasting the character, or progression of the monsoon timing and magnitude. Through a statistical categorization of historical observations, characteristics of subseasonal rainfall are grouped, or clustered by similarity of respective years, creating a statistical typology of monsoon rainfall. To our knowledge, such an approach based entirely on observed subseasonal statistics has not been previously attempted. This study further empirically examines the relationship between observed subseasonal extremes and agricultural yields in Niger [14].

The overarching research question that this study addresses is whether this statistical approach of clustering at seasonal timeseries is able to produce a distinct typology of monsoon rainfall progression. If so, then such a typology could potentially be used to identify precursors and drivers for prediction of the monsoon types. This approach could also be tested in other regions, with potentially large-scale implications. First, we outline the data and statistical methods employed in this study. Next, we show and discuss the results of the examinations, and then close with some further discussion and conclusions.

## Data and methods

This study employed the CHIRPSv2 [15] merged (station data and remotely sensed data) rainfall product to examine the historical evolution of dekadal rainfall during the southern Niger monsoon season over the 1981–2021 period. The study domain is defined as a box bound by: latitudes 11.5 to 16 degrees north; and longitudes 0.5 to 14 degrees east (see blue box S1 Fig in S1 File). As a representative example of station density within this domain in the post-2005 period, the locations of the 13 stations available in March of 2020 are also shown (bottom panel). The domain is spatially coherent, as the stations are well-distributed and timeseries are well-correlated in space during the 41-year May-September study period. This coherency lends confidence for using the spatial mean of the domain for timeseries analyses.

We employed k-means cluster analysis to statistically determine groups of similar years, based on each year’s subseasonal characteristics. After taking the domain average of rainfall for each dekad during the monsoon season, we arranged the data in two dimensions: 15 dekads for each season by 41 years. The clustering was performed using a squared Euclidean distance metric and the k-means algorithm to establish centroids. Ward’s linkage was also used to construct a hierarchical tree, and the silhouette and Calinski-Harabasz criteria were used to evaluate the clustering solutions. By determining the clusters in this way, we remove several potential biases. First, no a priori assumptions regarding the number of categories of rainfall are made (wet, normal and dry terciles, for example, have often been used in seasonal forecasts). Also, the normality or relative skewness of the clustered distributions, and particularly the sample size, or number of years in each respective cluster are not predetermined. All resulting clusters have a size or length of 15 dekads, and the number of clusters (groups of similar years) is optimized. To further quantify the ability to discriminate the resulting clusters of years from each other, we used a two-sample Kolmogorov-Smirnov test, based on a 99% confidence interval (alpha = .01) to compare each respective dekad subsequent to the clustering. Probability density functions of the clustered distributions were produced using a gamma fit.

## Results

Over the 1981–2021 period, the five-month May-September monsoon season is responsible for nearly all (>99%) of the domain’s annual rainfall. The climatological (1981–2021 mean) monthly progression of rainfall during the season is shown in S1 Fig of S1 File.

Based on the clustering criterion employed (see Methods), the optimal number of data clusters (groups of years with similar seasonal rainfall progression) was found to be two. The ability to discriminate between clusters two and three was markedly reduced, and cluster three was therefore determined to be insufficiently distinct. Cluster one (shown hereafter in blue) contains 14 years, and cluster two (red) is comprised of the remaining 27 years. See S1 Table in S1 File for a list of the years that fall into each cluster. Having divided the 41 years into the two groups, we now proceed to examine them in more detail.

**Fig 1:**
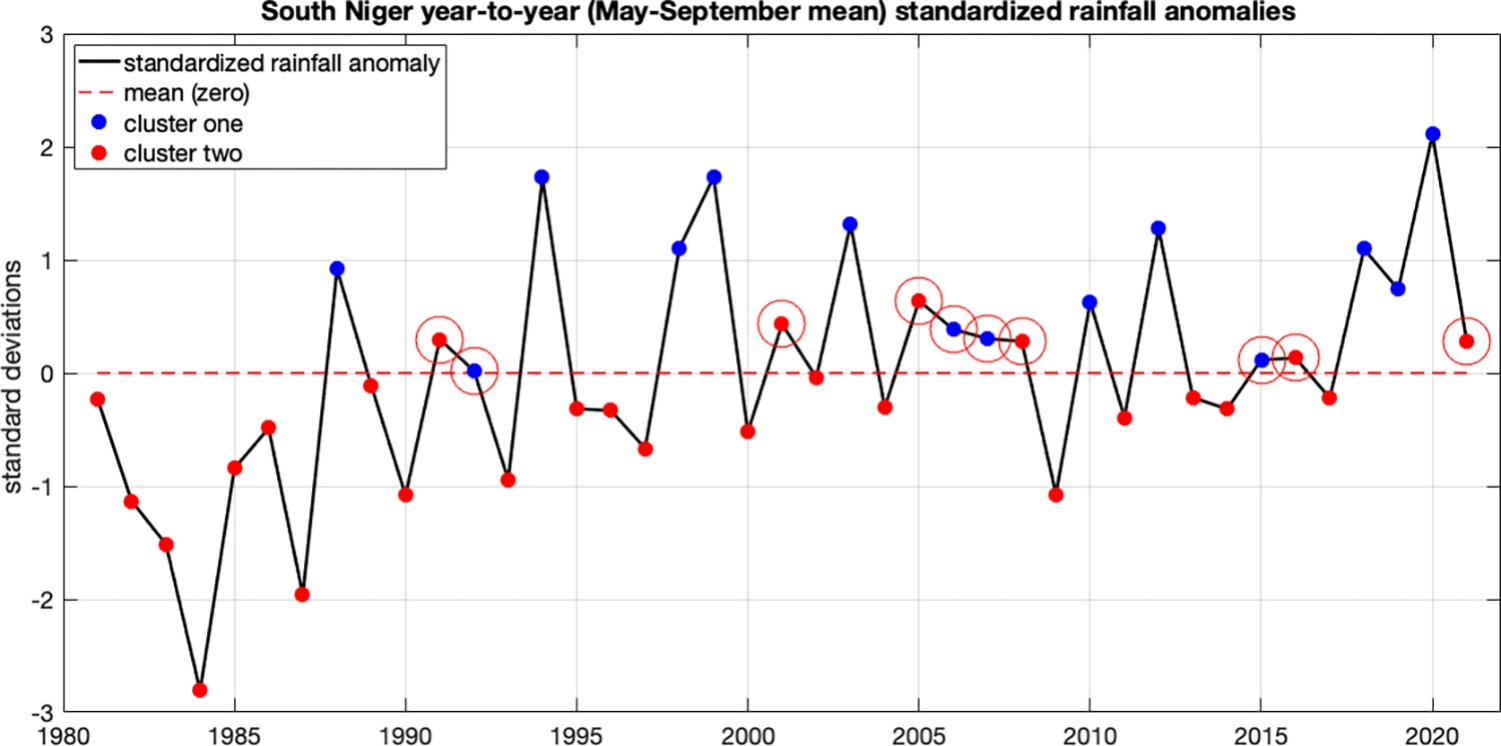
Standardized anomalies of seasonal (May to September) mean rainfall, 1981–2021, CHIRPSv2 data. Red and blue dots indicate respective clusters, based on seasonal rainfall progression.

First, in Fig 1 we show each year’s standardized seasonal mean rainfall (timeseries, in black), and compare it to its respective yearly cluster (blue or red), determined using each year’s subseasonal variability. Years apportioned to cluster one are denoted with blue dots, and years of cluster two are shown in red. We see that all of the cluster one years (blue) have positive yearly standardized anomalies. Most (21 of 27) of cluster two years (red) have negative yearly standardized anomalies. Certain years (1991, 2001, 2005, 2008, 2016 and 2021) exhibited positive yearly anomalies, yet were appropriated to the “wet” cluster (blue). Thus, while the annual anomalies and the clusters generally correspond, the clustering approach also captures subseasonal characteristics that are not necessarily reflected in the yearly anomalies. Some years that experience above normal total rainfall during the season are grouped with the red cluster due to their subseasonal characteristics rather than their seasonal rainfall total alone. Additionally, we see four years (1992, 2006, 2007 and 2015) during which subseasonal anomalies occurred that placed them in the blue cluster (typically “wet”), yet their seasonal means were near normal.

**Fig 2:**
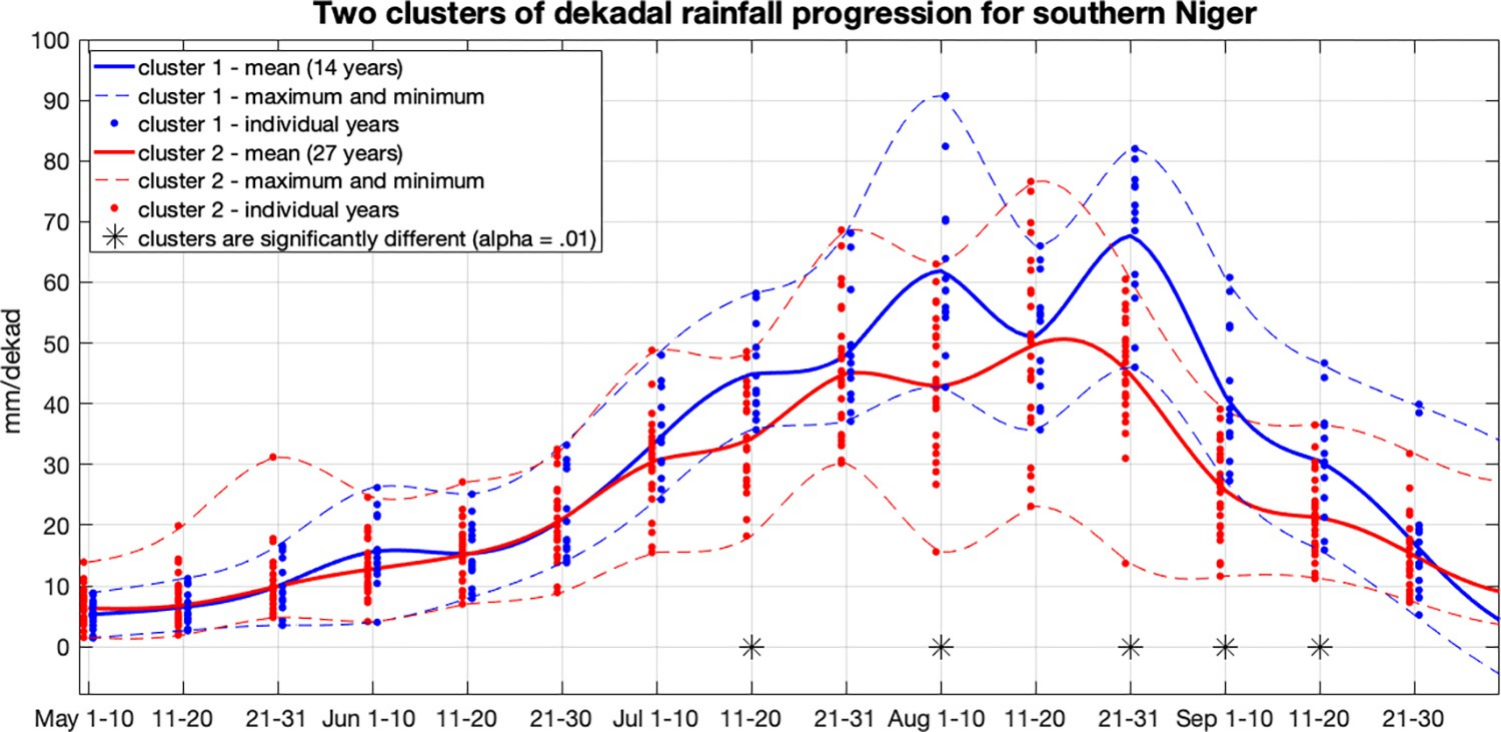
Progression of dekadal (ten-day) rainfall (May to September, 1981–2021). Two clusters of years are represented, shown in blue (14 years) and red (27 years). CHIRPSv2 dekadal rainfall product.

Fig 2 shows the dekadal mean rainfall for each year. In this way, we can see the spread of all years in each dekad. The years are divided into the two clusters (shown in blue and red). Dark blue and red lines represent the means of all years in each cluster, and dashed blue and red lines show the maximum and minimum years for each cluster. For the first two dekads in May, the spread in accumulated rainfall across all years ranges from 1.4mm in the lowest year to 13.9mm in the highest year, with an interannual variance over all years of 7.6mm. By late June, the range increases to between 8.9 mm and 33.2mm, and in July and August continue to increase to a maximum range of 15.6mm to 90.7mm during the first dekad in August. During the entire month of August, the observed ranges represent considerable uncertainty from year-to-year, with dekadal totals as low as 14mm (late August 1984) and as high as 90.7mm (early August 1994). The ranges of rainfall for each dekad decline through the remainder of the season. September is typically wetter on average than May and June, and exhibits larger interannual variability. The final two dekads of September demonstrated some years with >35mm, values that did not occur in the early monsoon months. Overall, outlier years (relative to each dekad’s historical distribution) occurred more frequently in August and September than in May, June or July, which exhibited a narrower range of uncertainty.

We used a two-sided Kolmogorov-Smirnov test to explore which dekads are most distinct when comparing the two clusters. This revealed five dekads in which the two groups are significantly (alpha = 0.01) dissimilar, and therefore most responsible for distinguishing between the two groups of years. These dekads are the second dekad in July, the first and last dekads in August, and the first two dekads in September, noted with asterisks below the timeseries (Fig 2). For the ten other dekads, the null hypothesis (that the two groups come from the same population) could not be rejected based on a 99% confidence interval. The difference between probability distributions of the two groups is particularly evident in the first and third dekads in August and the first dekad in September. Even for these dekads, however, there is notable overlap between the two groups, indicating uncertainty.

**Fig 3:**
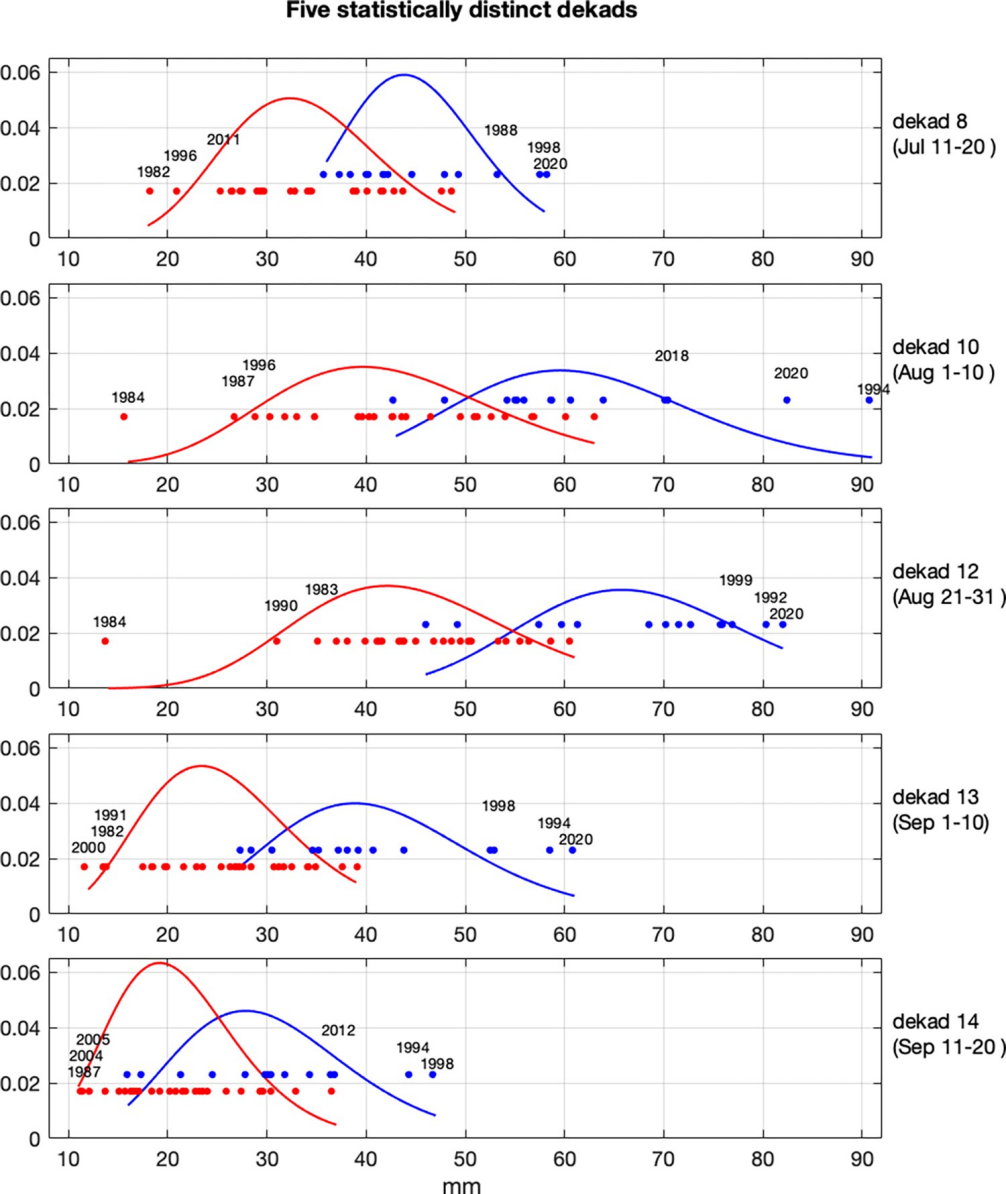
Probability density functions (based on a gamma fit) of the two clusters (blue and red) of dekadal (ten-day) rainfall during the May to September season (1981–2021, CHIRPSv2 data). Shown are the five dekads in which the clusters are significantly distinct from one another (alpha = 0.01).

The probability density functions of the clustered distributions for the five significant dekads are shown in Fig 3. As shown in Fig 3, the first and third dekads in August and the first dekad in September are the most distinct of the five, resulting in a narrower range of overlap between the two clusters. Also shown (Fig 3) are the five wettest years in the red cluster and the five driest years in the blue cluster for each dekad. The 2020 season as a whole was the wettest in the last 41 years (since the beginning of the CHIRPS data), with three of the five dekads during that season producing record highs of rainfall (also see Fig 2). The 1994, 1998 and 1999 years were also extremely wet, and to a lesser degree, 1988, 2003 and 2012.

During the blue (typically “dry”) clustered years that exhibited positive seasonal anomalies (1991, 2001, 2005, 2008, 2016 and 2021; see Fig 2), there were dry to very dry episodes prevalent during early and late August and early September that are characteristic of the blue cluster of years. This implies that subseasonal anomalies during these critical dekads are an important descriptor of the type of rainfall progression during a given monsoon season.

**Fig 4:**
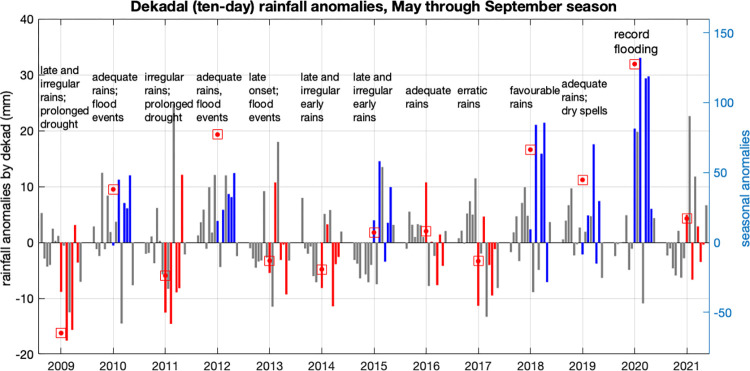
Rainfall anomalies by dekad (ten days), May-September season, CHIRPSv2 data. Significant dekads for blue and red clustered years are indicated.

The observed May-September dekadal rainfall anomalies from 2009–2021 (solid blue) are shown in Fig 4. The seasonal climatological cycle (the median value of each dekad) was removed for each year. The respective clusters for each year are denoted with blue and red dots below. The values representing each of the five significant dekads each year are also shown (black dots). Based on cooperative FAO and WFP inter-agency special reports on crop yields and food security [16], annotations are shown representing the relationship between reported rainfall and “bad” agricultural years during this period. These anecdotal reports indicate aspects of irregular rainfall during the rainy season that are reported to have adversely affected crop production, and are compared here against the rainfall data to look for consistencies. Over this period, each year has distinguishing characteristics with respect to its rainfall anomalies, such as extreme wet or dry dekads or persistent wet or dry episodes, that are reflected in both the meteorological data and the textual reports.

As shown in Fig 3, based on the 41 years of observations, rainfall extremes during dekads at the beginning of the rainy season represent a more modest range of uncertainty, whereas the extreme events that occurred during the middle of the season, particularly in August, represent a wide range of uncertainty.

These results reinforce those shown in Fig 3, indicating very little distinction between the two clusters of years during May and June. The distinction between the two optimal clusters of years is due almost entirely to the divergent trajectories of the two clusters beginning in July and building into August and September.

**Fig 5:**
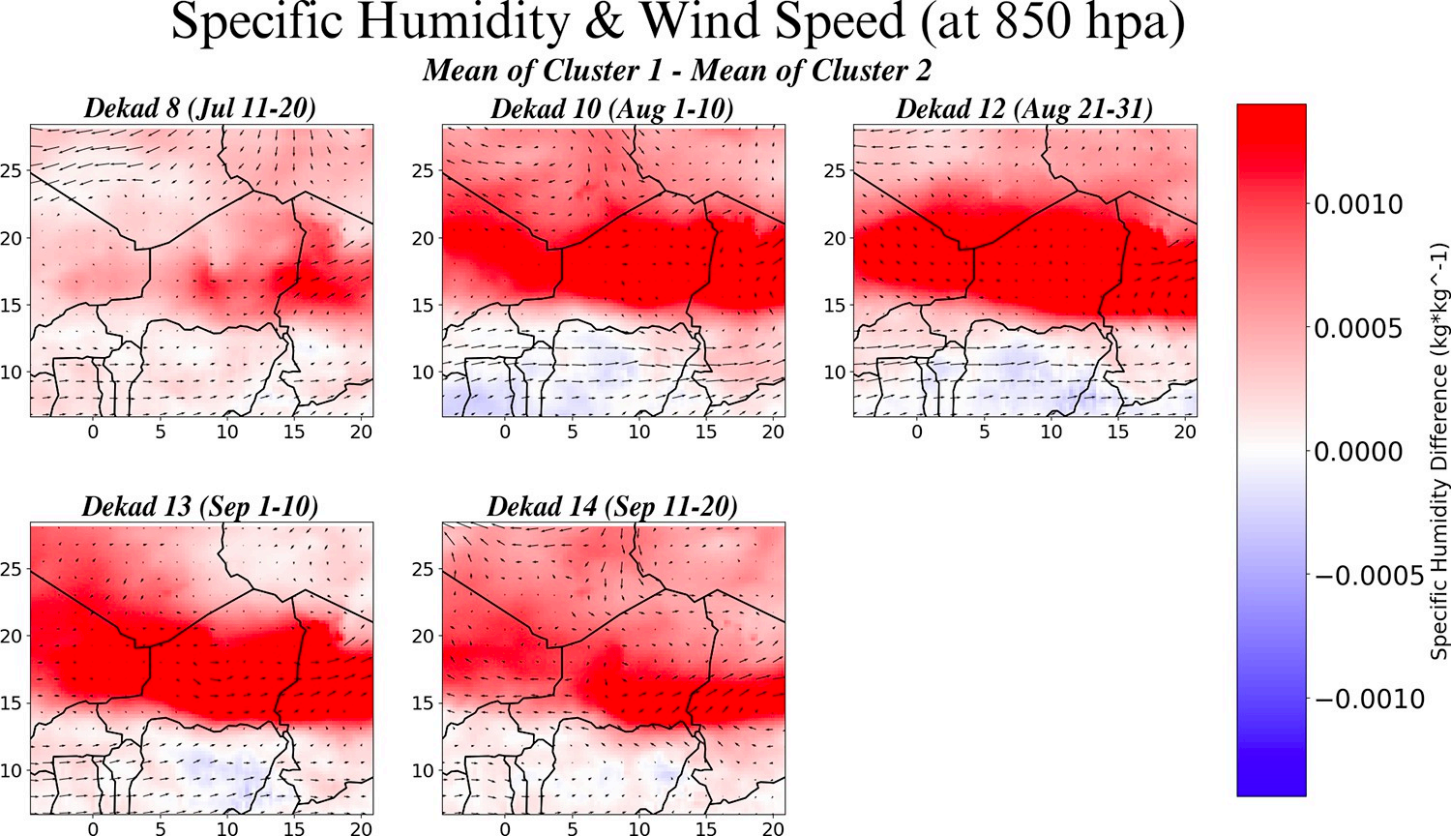
Low-level (850hPa) specific humidity and wind fields. Difference between composites of the two yearly clusters, for the five significant dekads. ERA5 reanalysis data.

The differences in the circulation anomalies associated with the two clusters of years for the five significant dekads are shown in Fig 5. The low-level (850hPa) specific humidity and wind fields (from the ERA5 reanalysis [17]) reveal distinct differences between the two clusters. Particularly evident is the large difference in the availability of low-level moisture present during cluster one years.

## Conclusions

Niger’s vulnerability is becoming increasingly acute. The country has seen a rise of more than 40% in both IDP and refugees (primarily from Nigeria and Mali) since 2019 (UNGHO). After record flooding in 2020, eight million people were under pressure from food insecurity during 2021, including 2.3 million severely affected (UNGHO). Gross cereal production in Niger decreased by 39% from 2020/21 to 2021/22 (FEWSNET West Africa). The Diffa region and the extreme south of the Maradi region, both affected by civil insecurity, will remain in the IPC (Integrated Food Security Phase Classification) Acute Food Insecurity Phase two (“Stress”) until September 2022, thanks to food assistance. The borders with Chad and Burkina Faso, however, will remain in Phase three (“Crisis”). The global acute malnutrition rate among children under five years of age rose to 12.7 percent in 2020, exceeding the World Health Organization (WHO) emergency threshold of 10 percent. This heightened exposure intensifies the need for more comprehensive coping and decision-making tools and strategies. The explorations presented here may offer some opportunity for gains with respect to better understanding and potentially improved prediction of subseasonal extremes that are known to be critically important for agricultural success.

These results demonstrate several key findings. Early in the season, particularly during May, anomalies from year-to-year are insufficiently large enough to effectively distinguish between them, for predictive purposes. This modest range of variability over all observed years implies that efforts to predict anomalies in the timing of monsoon onset in a binary way such as this, would be challenging. In essence, the most effective prediction of rainfall anomalies early in the season is the climatology, as deviations from the climatology are comparatively small.

Based on the clustering of years into two categories, five particular dekads during the season were found to be significantly distinct with respect to the two groups. The ability to distinguish one group of years from another demonstrates promise for utilizing these seasonal types, or trajectories, to identify key dynamic predictors or statistical precursors for enhanced prediction of within-season rainfall characteristics. Important questions remain to be answered with respect to the identification of such predictors (of both rainfall extremes and of agricultural yields) at a range of lead times, perhaps extending to the previous year.

Total seasonal rainfall alone is not an adequate metric to capture all “bad” years; some years that received what would be characterized as “normal” total rainfall over the course of the season experienced rainfall anomalies within the season that were detrimental to the agriculture. Reports confirmed that subseasonal anomalies such as “delayed onset,”“late rains”, “irregular” or “erratic” rains, flood events and dry spells all were reported to contribute to poor crop yields in different years, and in many cases two or more of these anomalies combined in a given year.

These investigations conclude that better use and incorporation of subseasonal rainfall variability statistics to aid in the advance identification of such subseasonal rainfall extremes could provide additional valuable information for decision-makers.

## Supporting information

S1 File 1) Monthly rainfall climatologies (mean of the 1981–2021 period), CHIRPSv2. The focus domain of this study is indicated by the blue box. Also shown (top panel) are the locations of the stations used by CHIRPSv2 in March 2020, for reference. 2) Two optimized clusters, or groups of similar years of rainfall progression.
(DOCX)

## References

[1] U.N. Global Humanitarian Overview, https://2021.gho.unocha.org.

[2] World Bank Data, https://data.worldbank.org/indicator/SP.DYN.TFRT.IN?locations=NE.

[3] FAO, https://www.fao.org/agriculture/ippm/projects/niger/en/.

[4] Colman et al. 2017 book chapter [from Parker D., and Diop-Kane M, Eds., 2017: Meteorology of Tropical West Africa: The Forecaster’s Handbook. Wiley-Blackwell, 496 pp. Google Scholar].

[5] ReliefWeb, https://reliefweb.int/country/ner.

[6] USAID, https://www.usaid.gov/climate/country-profiles.

[7] FEWS NET West Africa, https://fews.net/west-africa/niger.

[8] FAO, https://www.fao.org/countryprofiles/index/en/?iso3=NER.

[9] Vigaud N., Robertson A.W., Tippett M.K. and Acharya N., 2017. Subseasonal predictability of boreal summer monsoon rainfall from ensemble forecasts. Frontiers in Environmental Science, 5, p.67.

[10] Barnston A. G., Tippett M. K., Ranganathan M. and L’Heureux M. L. 2017: Deterministic skill of ENSO predictions from the North American Multimodel Ensemble. Clim. Dyn., 1–20. doi: 10.1007/s00382-017-3603-3 31929685 PMC6936341

[11] Giannini A., Ali A., Kelley C.P., Lamptey B.L., Minoungou B. and Ndiaye O., 2020. Advances in the lead time of Sahel rainfall prediction with the North American Multimodel Ensemble. Geophysical Research Letters, 47(9), p.e2020GL087341.

[12] Olaniyan E., Adefisan E.A., Oni F., Afiesimama E., Balogun A.A. and Lawal K.A., 2018. Evaluation of the ECMWF sub-seasonal to seasonal precipitation forecasts during the peak of West Africa monsoon in Nigeria. Frontiers in Environmental Science, p.4.

[13] Kumi N., Abiodun B.J. and Adefisan E.A., 2020. Performance evaluation of a subseasonal to seasonal model in predicting rainfall onset over West Africa. Earth and Space Science, 7(8), pp.e2019EA000928–T.

[14] Shukla S., Davenport F., Grace K., Tinni S., Ali A., Adoum A., et al. 2022, December. Using subseasonal climate variability at the community-level to advance place-based early warning systems in West Africa. In Fall Meeting 2022. AGU.

[15] Funk C., Peterson P., Landsfeld M., Pedreros D., Verdin J., Shukla S., et al. 2015. The climate hazards infrared precipitation with stations—a new environmental record for monitoring extremes. Scientific data, 2(1), pp.1–21. doi: 10.1038/sdata.2015.66 26646728 PMC4672685

[16] FAO, https://www.fao.org/giews/countrybrief/country/NER/pdf_archive/NER_Archive.pdf.

[17] Hersbach H., Bell B., Berrisford P., Hirahara S., Horányi A., Muñoz‐Sabater J., et al. 2020. The ERA5 global reanalysis. Quarterly Journal of the Royal Meteorological Society, 146(730), pp.1999–2049.

