# Supplementary figures and images for: A typology of subseasonal rainfall evolution during the southern Niger monsoon
Source: PLoS One. 2024 Apr 9;19(4):e0299771. doi: 10.1371/journal.pone.0299771 (PMC11003671; doi:10.1371/journal.pone.0299771)

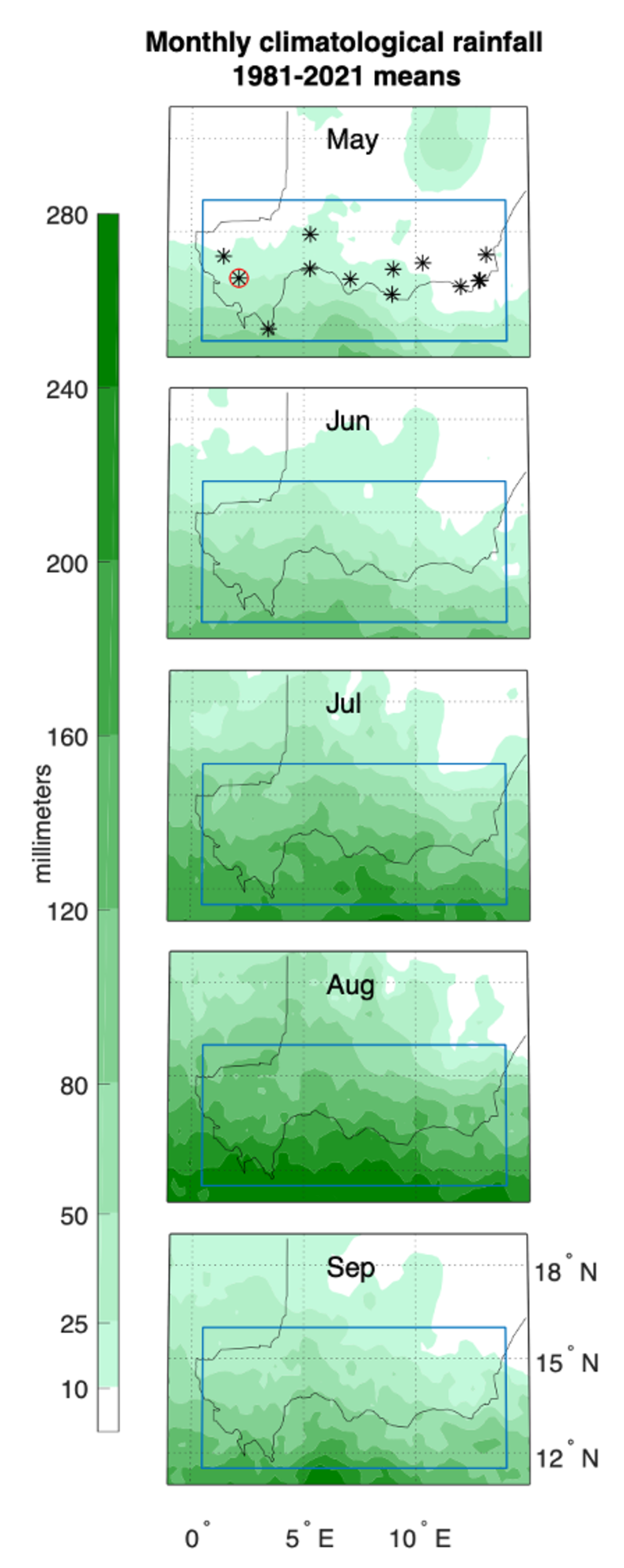


| Optimized clusters | | |
| --- | --- | --- |
|  | One | Two | |
|  | 1988 | 1981 | |
|  | 1992 | 1982 | |
|  | 1994 | 1983 | |
|  | 1998 | 1984 | |
|  | 1999 | 1985 | |
|  | 2003 | 1986 | |
|  | 2006 | 1987 | |
|  | 2007 | 1989 | |
|  | 2010 | 1990 | |
|  | 2012 | 1991 | |
|  | 2015 | 1993 | |
|  | 2018 | 1995 | |
|  | 2019 | 1996 | |
|  | 2020 | 1997 | |
|  |  | 2000 | |
|  |  | 2001 | |
|  |  | 2002 | |
|  |  | 2004 | |
|  |  | 2005 | |
|  |  | 2008 | |
|  |  | 2009 | |
|  |  | 2011 | |
|  |  | 2013 | |
|  |  | 2014 | |
|  |  | 2016 | |
|  |  | 2017 | |
|  |  | 2021 | |

Supplement: S1 File — The focus domain of this study is indicated by the blue box. Also shown (top panel) are the locations of the stations used by CHIRPSv2 in March 2020, for reference. 2) Two optimized clusters, or groups of similar years of rainfall progression. (DOCX) [file pone.0299771.s001.docx]
